# Research on the Bond Behavior of Preplaced Aggregate Concrete-Filled Steel Tube Columns

**DOI:** 10.3390/ma13020300

**Published:** 2020-01-09

**Authors:** Jing Lv, Tianhua Zhou, Qiang Du, Kunlun Li, Liangwei Jin

**Affiliations:** 1 School of Civil Engineering, Chang’an University, Xi’an 710061, China; zhouth@chd.edu.cn (T.Z.); 2017128074@chd.edu.cn (K.L.); jinlw@chd.edu.cn (L.J.); 2 School of Economics and Management, Chang’an University, Xi’an 710064, China; q.du@chd.edu.cn

**Keywords:** concrete-filled steel tube, preplaced aggregate concrete, bond behavior, push-out tests

## Abstract

In order to investigate the bond behavior of preplaced aggregate concrete-filled steel tube (CFT-PAC) columns and the difference of bond behavior between CFT-PAC columns and normal concrete-filled steel tube (CFT-NC) columns, a total of 11 columns were prepared and the push-out tests were conducted. The experimental parameters included the type of concrete (preplaced aggregate concrete and normal concrete), concrete strength (C40, C50 and C60), cross-section dimension (*D* = 219 mm, 299 mm and 351 mm) and the thickness of steel tube (*t* = 6 mm and 8 mm). The results indicated that the CTF-PAC columns had a similar load-slip curves with CFT-NC columns. The bond stresses of the CFT-PAC columns were higher than that of the PAC-NC columns at the same concrete strength. Increasing compressive strength of PAC increased the critical bond strength and bond strength of CFT-PAC columns. With an increase of the *L*/*D* ratio, both of the slip corresponding to peak load and bond strength of CFT-PAC columns exhibited an increasing trend. A rise in the *D*/*t* ratio led to a decrease in the bond stress of CFT-PAC columns and an increase in slip corresponding to the peak load of CFT-PAC columns. The proposed bond stress–slip relationship model considerably matched the bond stress–slip relationship of CFT-PAC columns.

## 1. Introduction

The concrete–filled steel tube (CFT) structure is one of the types of structures which is comprised of core concrete and steel tube. Due to the special construction technology, the constraint effect of core concrete by steel tube and the cooperative work between the core concrete and steel tube will be achieved [1,2]. Therefore, this structure has plenty of virtues in terms of greater carrying capacity, higher lateral stiffness, better ductility properties, much more efficient construction and easier fabricated construction [3,4,5,6,7]. Based on the above advantages, CFT structures have been widely used in industrial plants, long-span bridges, high-rise buildings and underground structures in recent years [8,9,10,11,12].

As one of the structural components of CFT structures, CFT columns play an important role in CFT structures systems. Plenty of studies have been conducted on the properties of the CFT column, including compressive behavior [13,14,15], bond behavior [16,17,18,19], seismic performance [20,21], fire resistance [22,23,24], and so on. Meanwhile, numerous different types of concrete have been also used in concrete-filled steel tubes, such as lightweight aggregate concrete [25,26], high-strength concrete [27], recycled aggregate concrete [28,29,30,31,32] and fiber-reinforced plastic (FRP)-confined concrete [33,34]. Utilization of different types of concrete in CFT will achieve disparate benefits. For example, using lightweight aggregate concrete in CFT is a benefit to reduce the self-weight of CFT, using high-strength concrete in CFT is another benefit to enhance the bearing ability of CFT and using recycled aggregate concrete in CFT is on more benefit to recycle the construction waste.

Preplaced aggregate concrete (PAC) is an eco-efficient concrete with a quite different preparation process from normal concrete (NC). The preparation process of NC is mixing all the raw materials together firstly and then casting them into the formworks, whereas, the preparation process of PAC is preplacing coarse aggregate in formworks and then injecting the grout mortar into the void between coarse aggregate by pumping. Compared with NC, in PAC, the content of coarse aggregate is higher while the content of the grout mortar is lower. Thus, a series of benefits will be inevitably acquired by the development of PAC, such as saving cement, increasing concrete stiffness and reducing concrete shrinkage as compared with NC [35,36,37,38,39,40].

On the basis of the above characteristic of PAC, combined with the recent popular construction method of the modular construction of columns in CFT strictures [41], it may be a good attempt to replace NC by PAC in CFT structures which will achieve a series of benefits, typically in reducing both the cement content and cost of CFT structures, enhancing the bond between core concrete and steel tube, increasing the stiffness of CFT structures, improving the pouring quality of core concrete inside CFT structures. However, very few researches have been reported in this area. Thus, it is necessary to conduct the investigations on the properties of CFT structures filled with PAC.

As one type of the most widely used CFT members, the CFT column filled with PAC (CFT-PAC) is selected as the research object in this paper. Generally, in order to promote and utilize CFT-PAC columns in engineering practices, a comprehensive understanding on the mechanical behavior of the CFT-PAC column is indispensable. However, as a novel structural member proposed in this research firstly, very little research is conducted on the mechanical behavior of the CFT-PAC column. Considering the bond behavior between core PAC and steel tube will significantly affect the mechanical behavior of the CFT-PAC column, and a better understanding on the bond behavior of the CFT-PAC column is a benefit to accurately analyze the mechanical behavior of the CFT-PAC column. Nevertheless, so far, there is very little existing research about the bond behavior of the CFT-PAC column. Despite the fact that the bond behavior of the CFT column has been investigated by numerous studies, there is still a gap between CFT and NC which will lead to a fact that using the bond behavior of the CFT-NC column to analyze the mechanical behavior of CFT-PAC may be inaccurate. Hence, the bond behavior of CFT-PAC column is needed to be studied.

The present research is focused upon the influence of different parameters on the bond behavior of CFT-PAC columns. The bond behaviors of CFT-PAC including load-slip curves, axial and lateral strain distributions, failure modes and bond stress are measured by push-out tests and discussed in detail. The considered parameters in this experiment include the type of concrete (PAC and NC), concrete strength (C40, C50 and C60), cross-sectional dimension (*D* = 219 mm, 299 mm and 351 mm), and thickness of the steel tube (*t* = 6 mm and 8 mm). All specimens are tested at the age of 28 days. Furthermore, the bond stress–slip relationship models for CFT-PAC are also proposed. The results from this research will provide the bond stress–stain relationships of the CFT-PAC columns, which will guide the design of CFT-PAC columns efficiently.

## 2. Materials and Methods

### 2.1. Test Specimens 

In this paper, a total of 11 specimens were designed and prepared to investigate the effect of structural parameters on the bond behavior of concrete-filled steel tube columns. The structural parameters were (a) type of concrete (PAC and NC); (b) concrete strength (C40, C50 and C60); (c) cross-sectional dimension (*D* = 219 mm, 299 mm and 351 mm); (d) thickness of steel tube (*t* = 6 mm and 8 mm). The details of the specimens are shown in Table 1. The *L* denoted the length of specimens, the *D* denoted the external diameter of specimens and the *t* denoted the thickness of the steel tube. The CFT-PAC and CFT-NC denoted that the steel tube was filled with PAC and NC, respectively.

### 2.2. Material Properties and Concrete Proportions

#### 2.2.1. Material Properties

The grade of steel utilized in this research was Q235. According to GB/T 228-2010 [42], the mechanical properties of steel, including yield strength *f_y_*, ultimate strength *f_u_* and elastic modulus *E_s_* were tested and the measured values were presented in Table 2. The representative values listed in Table 2 were the average value of three samples.

The cubic compressive strength and elastic modulus of PAC and NC were measured in compliance with GB/T 50081-2002 [43]. The specimen dimensions of cubic compressive strength and elastic modulus were 100 mm × 100 mm × 100 mm and 100 mm × 100 mm × 300 mm, respectively. All tests were conducted on three specimens, and the average value was used to be a representative value, as shown in Table 3. The testing age was 28 days.

#### 2.2.2. Concrete Proportions

**Figure 1:**
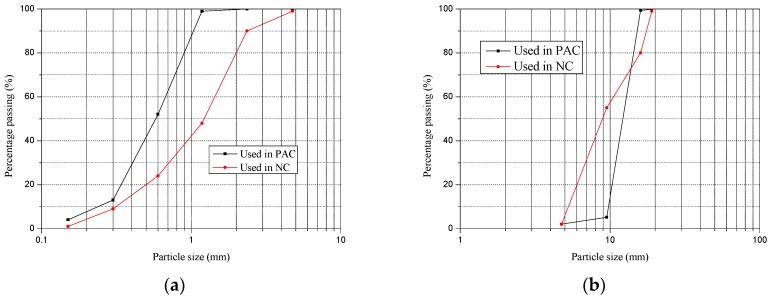
Particle size distribution of sand and coarse aggregate. (**a**) Sand, (**b**) Coarse aggregate.

A total of six mixtures were designed in this research, as presented in Table 3. All mixtures consisted of cement, sand, coarse aggregate, water reducer and water. The cement was Ordinary Portland Cement with a grade of 42.5 (GB 175-2007 [44]). The Chemical compositions and physical properties of this cement were summarized in Table 4. The water reducer was a polycarboxylate superplasticizer with a solid content of 40%. The water was from the tap (faucet). For PAC, the properties of the sand were an apparent density of 2610 kg/m^3^, modulus of fineness of 3.58, bulk density of 1450 kg/m^3^, maximum size under 2.36 mm and the particle size distribution as shown in Figure 1a. The properties of coarse aggregate were the crushing index 9.5%, loose bulk density 1360 kg/m^3^ and the apparent density 2520 kg/m^3^, while the particle size ranged from 4.75 mm to 19 mm, and the particle size distribution as presented in Figure 1b. For NC, the properties of coarse aggregate were an apparent density of 2625 kg/m^3^, modulus of fineness of 2.68, bulk density of 1430 kg/m^3^ and the particle size ranged from 0.15 mm to 4.75 mm, while the particle size distribution is as shown in Figure 1a. The properties of coarse aggregate were a crushing index of 8.6%, loose bulk density of 1420 kg/m^3^, apparent density of 2520 kg/m^3^, and its particle size ranged from 4.75 mm to 19 mm, while the particle size distribution is as presented in Figure 1b.

### 2.3. Preparation of Specimens 

**Figure 2:**
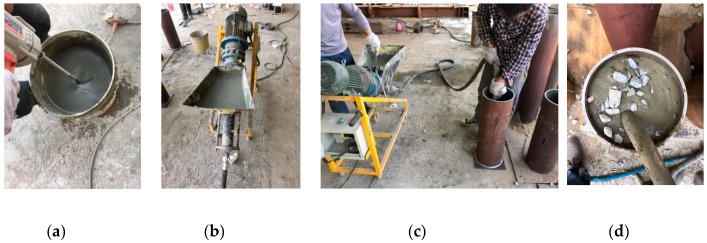
Preparation process of preplaced aggregate concrete-filled steel tube (CFT-PAC) specimens. (**a**) Preparation of grouting mortar; (**b**) Grouting device; (**c**) Grouting process; (**d**) Grouting effect.

The cold-formed circular steel tubes used in this research were cut according to the required sizes. For CFT-NC columns, the concrete was mixed firstly and then was filled into the steel tubes with vibration. For CFT-PAC columns, the grouting mortar was prepared firstly, then the coarse aggregate was cast into the steel tubes in layer, and finally the grouting mortar was injected into the void between coarse aggregate from bottom to top by pumping (preparation process as shown in Figure 2). The layered height of the coarse aggregate filled into the steel tubes was about 200 mm. The steel–concrete interface length for CFT-NC and CFT-PAC were all approximately 50 mm shorter than the length of the steel tubes. All specimens were cured for 28 days in an indoor environment with 20 ± 5 °C and RH > 95%.

### 2.4. Experimental Setup

**Figure 3:**
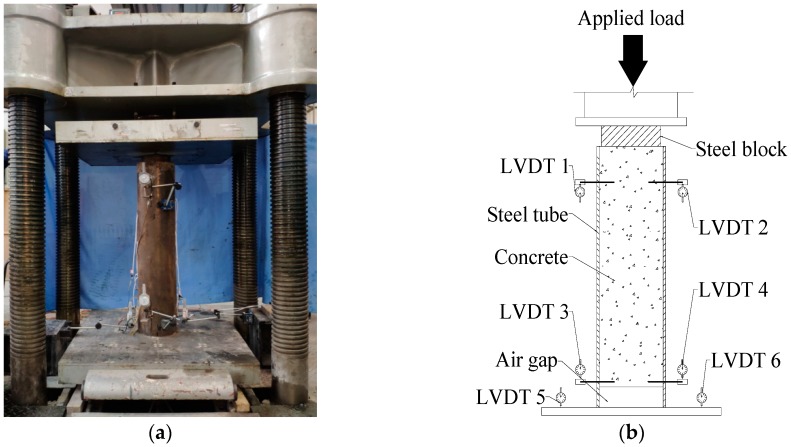
Experimental apparatus. (**a**) Test setup; (**b**) Placement of LVDTs.

**Figure 4:**
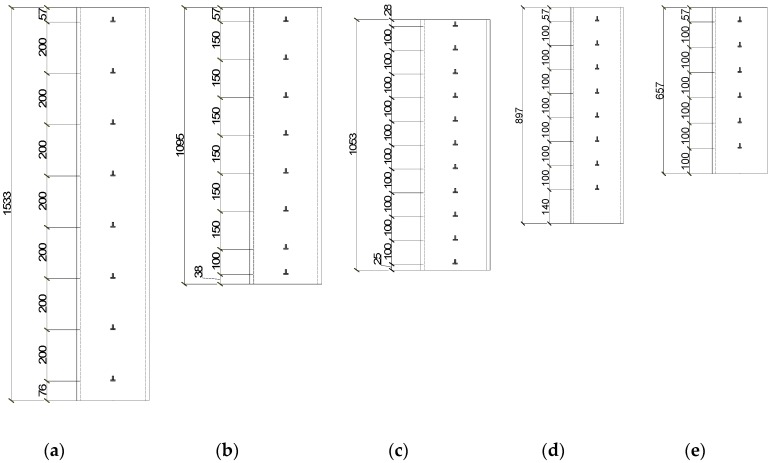
Location of strain gauge on the steel tube: (**a**) CFT-PAC-8; (**b**) CFT-PAC-7; (**c**) CFT-PAC-6; (**d**) CFT-PAC-5; (**e**) CFT-PAC-1~CFT-PAC-4, CFT-NC-1~CFT-NC-3.

In this research, the push-out tests were conducted to detect the bond behavior of CFT-PAC and CFT-NC. The experiment apparatus was presented in Figure 2. A 5000 kN computer-controlled electro-hydraulic servo universal testing machine was used to carry out the push-out tests. Prior to the tests, the specimen was vertically placed at the center of the testing machine. The loaded end was on the top and the free end was on the bottom. A steel pad placed between the upper platen of the testing machine and the specimen was used to make sure that the load was only applied on the concrete and the concrete could be pushed downward during testing. Two linearly varying displacement transducers (LVDTs) were installed at each end to measure the relative slip between concrete and steel tube (as shown in Figure 3). LVDT 1 and LVDT 2 were arranged at a distance of 100 mm from the upper surface of specimen. LVDT 3 and LVDT 4 were arranged at a distance of 100 mm from the bottom surface of specimen. Two LVDTs were installed on the bottom platen of the testing machine to measure the displacement of this same testing machine. A series of strain gauges were affixed on the front and back sides of the steel tubes to measure the lateral and axial strains along the length of the steel tube (as shown in Figure 4). The representative strain value of each point was acquired from the mean value of the front and back sides. For all specimens, the loading rate was at a constant rate of 0.02 mm/s. The test was terminated until the loading had little change while the slip increased dramatically.

The critical bond stress and bond strength could be calculated by the following formulas:

(1) $$\tau_{s}=\frac{P_{s}}{\pi DL}$$

(2) $$\tau_{u}=\frac{P_{u}}{\pi DL}$$

where *τ_s_* is the critical bond strength (MPa); *τ_u_* is the bond strength (MPa); *P_s_* is the critical load (N); *P_u_* is the ultimate load (N).

## 3. Results and Discussion

### 3.1. Load-slip Curves and Failure Modes

**Figure 5:**
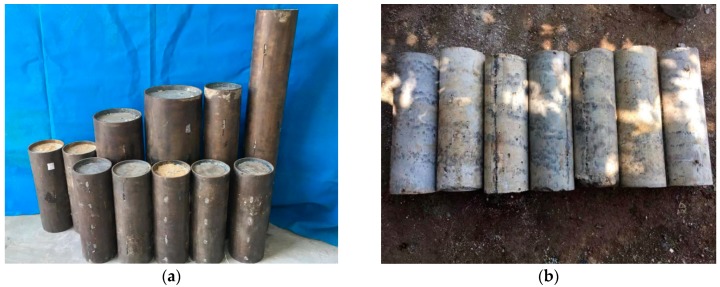
Typical failure mode of specimens. (**a**) Failure mode of all specimens; (**b**) The core concrete taken out from partial CFT-PAC columns.

Figure 5 presented the typical failure modes of specimens. A similar failure mode between different specimens indicated that the structural parameters had small effect on the failure modes of CFT-PAC and CFT-NC. For all specimens, at the initial stage of loading, no visible slip was observed between core concrete and steel tube. When the loading reached to approximately 70% of the bond failure load, a noise was heard and a little slip was noticed at the loading end. Then, with a continuing increase of applied load, the slip was noticed at the free end. When the loading reached to the bond failure load, the loading had no longer increased, while the slip increased dramatically. After bond-slip tests, the core concrete was removed from the steel tube. The compact surface of core concrete for all specimens demonstrated that the compact pouring effect of PAC could be achieved in steel tubes.

**Figure 6:**
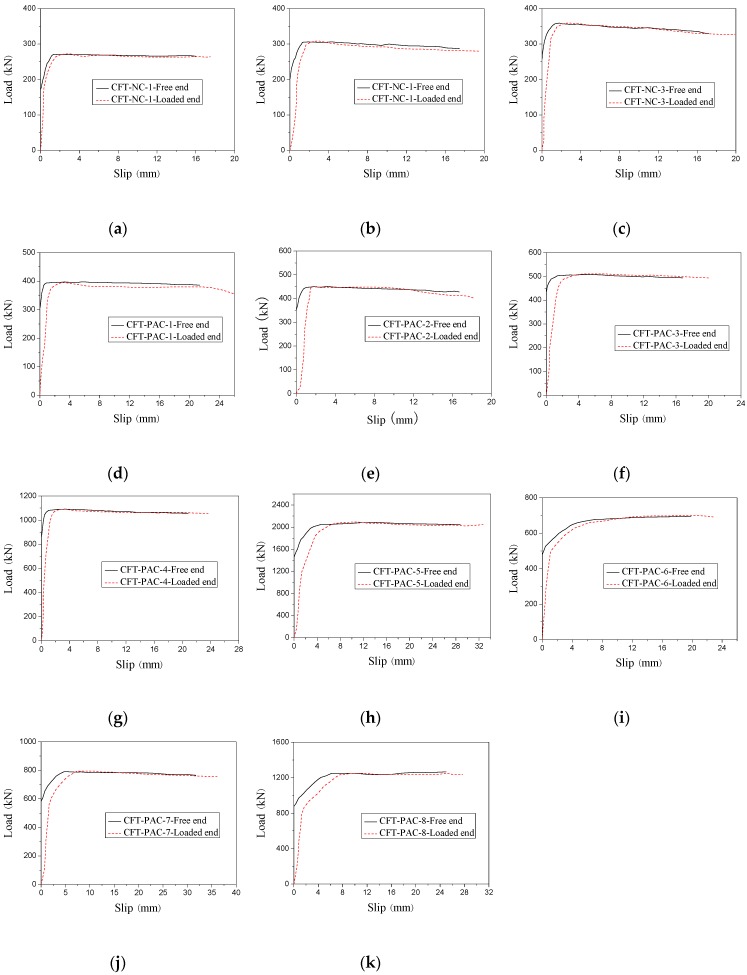
Load-slip curves at loading end and free end for specimens. (**a**) CFT-NC-1; (**b**) CFT-NC-2; (**c**) CFT-NC-3; (**d**) CFT-PAC-1; (**e**) CFT-PAC-2; (**f**) CFT-PAC-3; (**g**) CFT-PAC-4; (**h**) CFT-PAC-5; (**i**) CFT-PAC-6; (**j**) CFT-PAC-7; (**k**) CFT-PAC-8.

Figure 6 showed the load-slip curves of CFT-PAC and CFT-NC at the free end and loading end for all specimens. It could be noticed that different specimens had a similar shape of load-slip curves at the free end and loaded end. The displacement of the free end was selected to represent the relative slips between steel tube and the core concrete. Before critical load, the slip at the free end was inappreciable while the slip at the loaded end increased with the rise of load. After critical load, the slip at both of the free end and loaded end increased with the rise of load. When the load exceeded the bond load, the slip at both of the free end and the loaded increased continually while the load basically remained invariable. The load-slip curves could be divided into three stages: before critical load, critical load to bond load and after bond load, which was similar to the load-slip curves of normal concrete-filled steel tubes [16], manufactured sand concrete-filled steel tube [45] and recycled aggregate concrete-filled steel tube [30]. Before critical load, the bond resistance was mainly provided by chemical adhesion. At this stage, the applied load was resisted by chemical adhesion and the slip was not obvious. With increasing of applied load, the chemical adhesion declined gradually while the slip became noticeable, and the applied load reached to the critical bond. Then the specimens fell into the stage of critical load to bond load. At this stage, the friction and mechanical interlocking played an important role in the resisting of applied load. As the applied load increased, the bond resistance supplied by friction reduced while the bond resistance supplied by mechanical interlocking increased. Additionally, the slip increased continuously. When the applied load reached to the peak load, the applied load basically remained unchanged while the slip increased evidently. At this stage, the bond resistance was provided by the combination of residual friction and mechanical interlocking.

### 3.2. Axial and Lateral Strain Distributions

**Figure 7:**
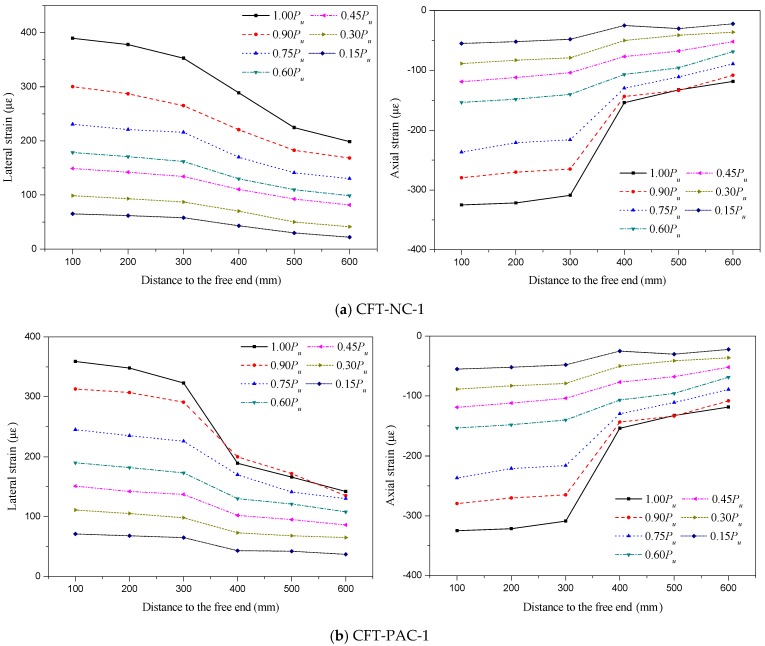

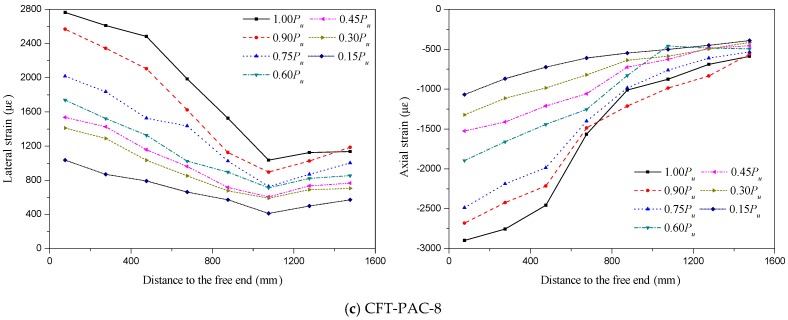
Typical lateral strain and axial strain distribution of the steel tube along the height. (**a**) CFT-NC-1; (**b**) CFT-PAC-1; (**c**) CFT-PAC-8.

The typical axial and lateral strains distribution of steel tube along height were shown in Figure 7. In these figures, the strain is the ordinate, the distance to the free end is represented by the abscissa. All specimens had a similar variation tendency on the axial and lateral strains distribution of steel tube along height, so the experimental results of CFT-NC-1, CFT-PAC-1 and CFT-PAC-8 were selected to analyze the axial and lateral strains distribution of the steel tube along height. As shown, at the initial stage of loading, the axial and lateral strains were all relatively small and were approximately linear with the distance to the free end. It indicated that the deformations at core concrete and steel tube were synchronous, where no obvious slip was occurred between core concrete and steel tube. With the increasing of applied load, the difference values of strain between the free end and loaded end became larger, and especially the applied load exceeded about 70% of the peak load. It indicated that the slip would occur when the applied load exceeded about 70% of the peak load which was verified by the test phenomena. The lateral strain and axial strain were all significantly affected by the location of strain. The higher the distance to the free end was, the smaller the lateral strain and axial strain would be. 

### 3.3. Influence of Different Parameters

#### 3.3.1. Effect of Concrete Strength and Concrete Type

**Figure 8:**
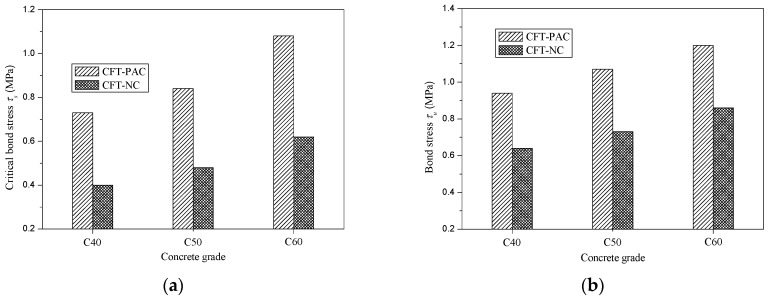
Variation of critical bond stress (*τ_s_*) and bond stress (*τ_u_*) with concrete strength and concrete type. (**a**) Critical bond stress (*τ_s_*); (**b**) Bond stress (*τ_u_*).

Figure 8 presented the variation of critical bond stress (*τ_s_*) and bond stress (*τ_u_*) with concrete strength and concrete type. Compared with CFT-NC, the critical bond stress (*τ_s_*) and bond stress (*τ_u_*) of CFT-PAC were larger at the same concrete grade. For critical bond stress (*τ_s_*), replacing NC by PAC in CFT would lead to an increment of 82.5% for C40, 75.0% for C50 and 67.7% for C60. Meanwhile, for bond stress (*τ_u_*), replacing NC by PAC in CFT would lead to an increment of 46.9% for C40, 46.6% for C50 and 39.5% for C60. The higher critical bond stress (*τ_s_*) and bond stress (*τ_u_*) of CFT-PAC might be mainly due to the lesser shrinkage strain of PAC as compared to NC. Because of the higher coarse aggregate content and lower cement content, the shrinkage strain of PAC was less than that of NC [36]. At the same concrete strength, the chemical adhesion, friction and mechanical interlocking between PAC and steel tube would be greater than that between NC and steel tube. Thus, the critical bond stress (*τ_s_*) and bond stress (*τ_u_*) of CFT-PAC was higher than that of CFT-NC at the same concrete strength. It indicated that applying PAC in CFT was beneficial to enhance the critical bond stress (*τ_s_*) and bond stress (*τ_u_*) of CFT. With the concrete strength increased, the critical bond stress (*τ_s_*) and bond stress (*τ_u_*) of CFT-PAC added that which was in accordance with the variation of CFT-NC. 

#### 3.3.2. Effect of Cross-Section Geometry

**Figure 9:**
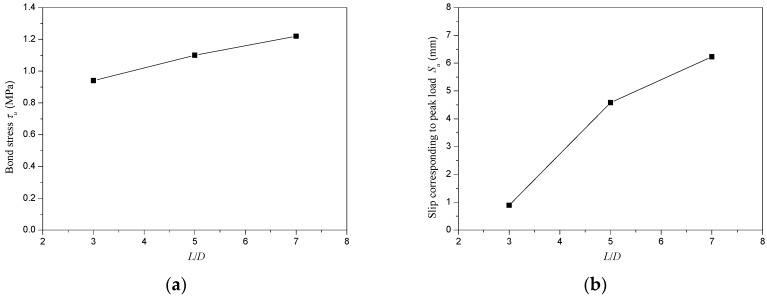
Variation of bond stress (*τ_u_*) and slip corresponding to peak load (*S_u_*) with *L*/*D* ratio. (**a**) Bond stress (*τ_u_*); (**b**) Slip corresponding to peak load (*S_u_*).

Figure 9 shows the variation of bond stress (*τ_u_*) and slip corresponding to peak load (*S_u_*) with the *L*/*D* ratio. In this paper, the *L*/*D* ratios were changed by varying the length of steel tube while maintaining the diameter of steel tube invariable. It could be seen that a rise in *L*/*D* ratio would lead to a slightly increase in bond stress (*τ_u_*) of CFT-PAC which was in accordance with CFT-NC columns [35]. Nevertheless, the slip corresponding to the peak load (*S_u_*) had an obvious augment as the *L*/*D* ratio increased. The slip corresponding to the peak load (*S_u_*) at the *L*/*D* ratio of 7 was approximately 7 times of the slip corresponding to peak load (*S_u_*) at *L*/*D* ratio of 3.

**Figure 10:**
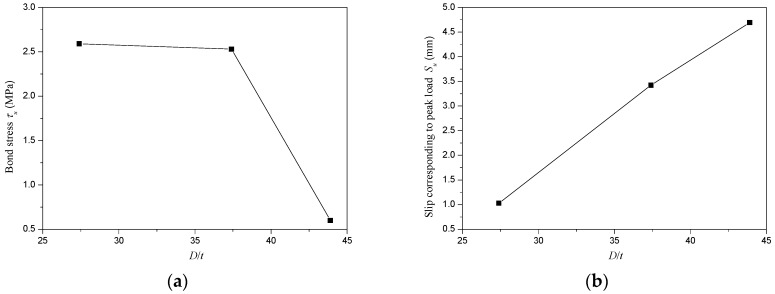
Variation of bond stress (*τ_u_*) and slip corresponding to peak load (*S_u_*) with *D*/*t* ratio. (**a**) Bond stress (*τ_u_*); (**b**) Slip corresponding to peak load (*S_u_*).

Figure 10 presented the variation of bond stress (*τ_u_*) and slip corresponding to peak load (*S_u_*) with *D*/*t* ratio. In this research, the *D*/*t* ratios were changed by varying the diameter of steel tube while maintaining the thickness of steel tube invariable. The bond stress (*τ_u_*) reduced with the increase of *D*/*t* ratio. A reduction of 2.3% in bond stress (*τ_u_*) was obtained as the *D*/*t* ratio augmented from 27.4 to 37.4, while a reduction of 76.3% in bond stress (*τ_u_*) was obtained as the *D*/*t* ratio increased from 37.4 to 43.9. It meant that if the *D*/*t* ratio exceeded 37.4, increasing *D*/*t* ratio would cause a drastic reduction of bond stress (*τ_u_*) which might significantly affect the bond-slip behavior of CFT-PAC. This was mainly because when the diameter of external diameter of steel tubes exceeded 299 mm, increasing the diameter of external diameter of steel tubes led to a drastic increase in the shrinkage strain of core concrete. The higher shrinkage strain of core concrete would reduce the adhesion between core concrete and steel tube, and then would decrease the bond stress of CFT-PAC. The slip corresponding to the peak load (*S_u_*) increased almost linearly with the *D*/*t* ratio, as shown in Figure 10. The observation of the variation tendency of bond stress (*τ_u_*) and slip corresponding to peak load (*S_u_*) of CFT-PAC with *D*/*t* ratio were similar to the circular steel tube filled with other types of concrete [16,30,45]. 

### 3.4. Bond Stress–Slip Relationship Model

**Figure 11:**
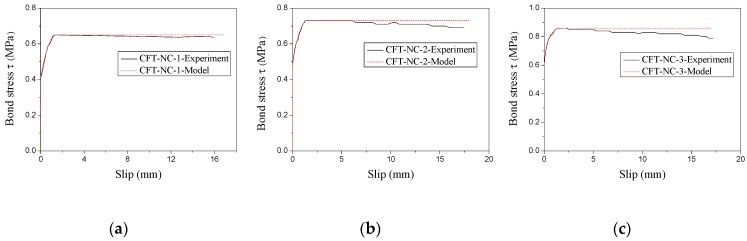

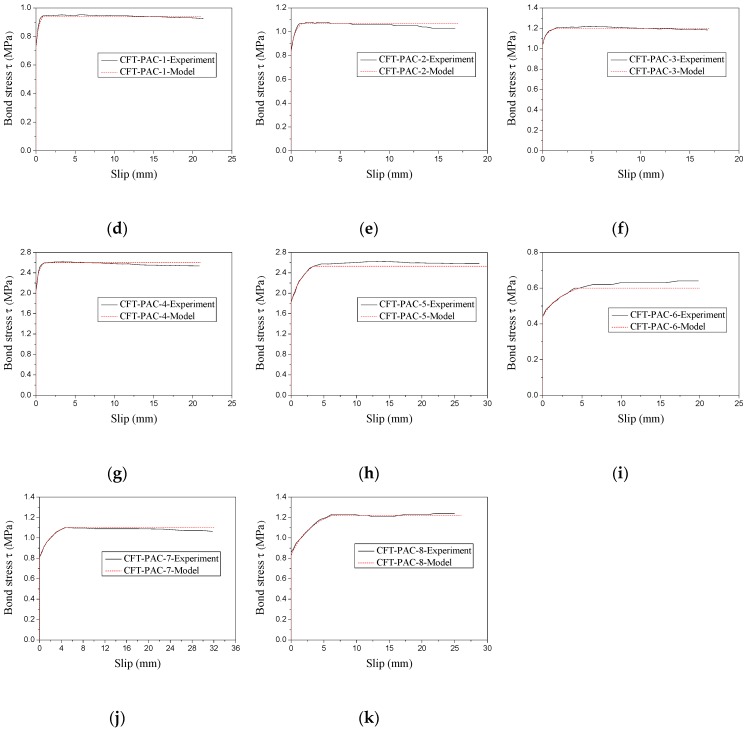
Comparison between testing curves and fitting curves. (**a**) CFT-NC-1; (**b**) CFT-NC-2; (**c**) CFT-NC-3; (**d**) CFT-PAC-1; (**e**) CFT-PAC-2; (**f**) CFT-PAC-3; (**g**) CFT-PAC-4; (**h**) CFT-PAC-5; (**i**) CFT-PAC-6; (**j**) CFT-PAC-7; (**k**) CFT-PAC-8.

On the basis of the experimental load-slip curves (as shown in Figure 6), the bond stress–slip curves of CFT-PAC at the free end could be ascertained as displayed in Figure 11. It could be found that bond stress–slip curves could be roughly divided into three branches: a branch with no slip, a branch which slip increased with rise of load, and a branch which load basically remained unchanged as the slip increased. As similar stress–slip curves had been reported on the CFT columns [46] and MS-CFT columns [45], the bond stress–slip relationship model for CFT columns and MS-CFT columns might be also utilized to depict the bond stress–slip relationship of CFT-PAC. In this research, an attempt to establish the relationship between the bond stress and slip of CFT-PAC following the existing bond stress–slip relationship of MS-CFT columns were given as follows:

(3) $$S=0\;(0\leq \tau <\tau_{s})$$

(4) $$\tau =\tau_{s}+\frac{S}{aS+b}\;(0\leq S\leq S_{u})$$

(5) $$a=\frac{S_{u}-2S_{0.5}}{\left(\tau_{u}-\tau_{s})(S_{u}-S_{0.5}\right)}$$

(6) $$b=\frac{S_{u}S_{0.5}}{\left(\tau_{u}-\tau_{s})(S_{u}-S_{0.5}\right)}$$

(7) $$\tau_{0.5}=0.5(\tau_{u}+\tau_{s})$$

(8) $$\tau =\tau_{u}\;(S_{u}<S)$$

where *S* is the slip at the free end (mm); *a* and *b* are parameters of nonlinear branch; *S_0.5_* is the slip corresponded to *τ_0.5_* at the free end (mm).

Figure 11 showed the comparison of the bond stress–slip curves of CFT-PAC obtained from experiment and fitting. As seen, the fitting curves of bond stress–slip curves of CFT-PAC agreed well with experimental results approximately. It indicated that the proposed relationship model could be utilized to describe the bond stress–slip relationship of CFT-PAC. It should be also noticed that the parameters *τ_u_*, *τ_s_*, *S_u_* and *S_0.5_* used in Equation (3)~Equation (8) were affected by numerous factors. A further research on the influence factors of the above parameters should be carried out and the prediction model of the bond stress–slip relationship of CFT-PAC was necessary to establish.

## 4. Conclusions

In this paper, a series of tests were conducted to evaluate the bond behavior of CFT-PAC. Base on the experimental results, a comparison of bond behavior between CFT-PAC and CFT-NC were conducted, meanwhile the failure mode and influence factors of bond behavior of CFT-PAC were analyzed. In addition, the bond stress–slip relationship mode of CFT-PAC was proposed. The main conclusions could be summarized as follows:

➢ The CTF-PAC columns had a similar load-slip curves with CFT columns filled with NC, manufactured sand concrete and recycled aggregate concrete. The bond stress of CFT-PAC was higher than that of PAC-NC at the same concrete strength.
➢ The different influence factors had effect on critical bond strength, bond strength and slip corresponding to peak load of CFT-PAC: (i) the critical bond strength and bond strength of CFT-PAC columns increased with the compressive strength of PAC added; (ii) the *L*/*D* ratio had a slight effect on the bond strength of CFT-PAC, but slip corresponding to peak increased obviously with the *L*/*D* ratio; (iii) the *D*/*t* ratio increased from 27.4 to 37.4 led to a reduction of 2.3% in the bond stress of CFT-PAC, while the *D*/*t* ratio increased from 37.4 to 43.9 led to a reduction of 76.3% in bond of CFT-PAC.
➢ The slip corresponding to the peak load of CFT-PAC increased almost linearly with the *D*/*t* ratio.
➢ The proposed bond stress–slip relationship model was suitable to describe the bond stress–slip relationship of CFT-PAC. A further research on the influence factors of parameters *τ_u_*, *τ_s_*, *S_u_* and *S_0.5_* was necessary to carried out to establish a prediction model for bond stress–slip relationship of CFT-PAC.

## Figures and Tables

**Table 1:** Details of specimens.

Specimen ID	*L* (mm)	*D* (mm)	*t* (mm)	*D*/*t*	Concrete Grade
**CFT-PAC-1**	657	219	6	36.5	C40
**CFT-PAC-2**	657	219	6	36.5	C50
**CFT-PAC-3**	657	219	6	36.5	C60
**CFT-PAC-4**	657	219	8	27.4	C40
**CFT-PAC-5**	897	299	8	37.4	C40
**CFT-PAC-6**	1053	351	8	43.9	C40
**CFT-PAC-7**	1095	219	6	36.5	C40
**CFT-PAC-8**	1533	219	6	36.5	C40
**CFT-NC-1**	657	219	6	36.5	C40
**CFT-NC-2**	657	219	6	36.5	C50
**CFT-NC-3**	657	219	6	36.5	C60

**Table 2:** Mechanical properties of steel.

Grade of Steel	*D* (mm)	*t* (mm)	*f_y_* (MPa)	*f_u_* (MPa)	*E_s_* (MPa)
**Q235**	219	6	340	510	2.05 × 10^5^
		8	332	492	2.08 × 10^5^
	299	8	341	503	2.06 × 10^5^
	315	8	343	511	2.06 × 10^5^

**Table 3:** Mix proportions and properties of Preplaced aggregate concrete (PAC) and normal concrete (NC).

Concrete Grade	Weight per Cubic Meter (kg/m^3^)					W/B	Elastic Modulus (GPa)	Cubic Compressive Strength (MPa)
	Cement	Sand	Coarse Aggregate	Water Reducer	Water			
**C60 (PAC)**	385	578	1360	5.78	116	0.30	44.1	65.7
**C50 (PAC)**	330	660	1360	4.98	100	0.30	42.1	57.2
**C40 (PAC)**	310	620	1360	2.48	124	0.40	40.2	44.7
**C60 (NC)**	500	660	1080	7.50	160	0.32	38.3	67.1
**C50 (NC)**	470	580	1180	7.05	170	0.36	35.8	56.8
**C40 (NC)**	420	570	1270	4.20	185	0.44	34.1	46.4

**Table 4:** Chemical compositions and physical properties of cement.

Chemical Analysis (%)	Cement
**CaO**	60.32
**SiO_2_**	22.34
**Al_2_O_3_**	4.55
**Fe_2_O_3_**	4.18
**MgO**	2.05
**SO_3_**	2.87
**K_2_O**	0.51
**Na_2_O**	0.41
**Loss on ignition**	2.77
**Specific gravity**	3.13
**Fineness (m^2^/kg)**	334
